# Influence of Donor Cells on the Efficiency of Cloned Ỉ Pig Production

**DOI:** 10.3390/ani16213384

**Published:** 2026-10-27

**Authors:** Au Thi Hoang, Vu Thi Thu Huong, Huu Xuan Quan, Hieu Trung Phan, Đat Van Le, Huong Thi Nguyen, Huong Le Thi Nguyen, Nhung Tuyet Thi Nguyen, Yen Kim Thi Pham, Duc Viet Phan, Van Anh Thi Nguyen, Nhien Thi Nguyen, Lan Doan Pham, Van Khanh Nguyen

**Affiliations:** 1 Key Laboratory of Animal Cell Technology, Vietnam Institute of Animal and Veterinary Sciences, Hanoi 12513, Vietnam; hoanghaiauqp@gmail.com (A.T.H.); vuhuongvcn8278@gmai.com (V.T.T.H.); qxhtntd@gmail.com (H.X.Q.); phanhieu2323@gmail.com (H.T.P.); vandat230696@gmail.com (Đ.V.L.); huongnguyentntd@gmail.com (H.T.N.); nguyenlehuongnias@gmail.com (H.L.T.N.); nhungniah@gmail.com (N.T.T.N.); phamthikimyen100696@gmail.com (Y.K.T.P.); duc10ck45@gmail.com (D.V.P.); phongtntdcntbdv@gmail.com (L.D.P.); 2 Faculty of Biomedical Science, Phenikaa University, Hanoi 12113, Vietnam; anh.nguyenthivan1@phenikaa-uni.edu.vn; 3 Faculty of Biotechnology, Vietnam National University of Agriculture, Hanoi 12406, Vietnam; ntnhien@vnua.edu.vn

**Keywords:** somatic cell nuclear transfer, Ỉ pig cloning, embryo transfer, cell passage number, cell line and donor cell sex

## Abstract

In this study, we investigated the effects of donor cell on the efficiency of producing cloned Ỉ pigs, an endangered Vietnamese breed. Cloned Ỉ pig embryos were generated using somatic cell nuclear transfer technology (SCNT). The results demonstrated that pregnancy, farrowing, and piglet production rates of Ỉ pig fibroblasts (ID:6004) at the 5th and 6th passages were significantly higher compared with those observed at the 4th and 7th passages. Under the conditions of this study, no significant effect of donor cell sex or cell lines on the efficiency of cloned Ỉ pig embryo transfer was observed. The pregnancy, farrowing, and piglet production rates did not differ significantly between donor cell groups of male and female origin (*p* > 0.05). Similarly, no significant differences were observed in pregnancy rates, farrowing outcomes, or one-week survival of live cloned piglets among the three donor cell lines (IDs: 6004, 9154, and 9157) (*p* > 0.05). These findings contribute to improving the efficiency of the somatic cell nuclear transfer (SCNT) technique in efforts to conserve the critically endangered Vietnamese Ỉ pig.

## Simple Summary

Somatic cell nuclear transfer (SCNT) represents an important technology for the preservation of pigs; however, its limited efficiency constrains wider application. In the present study, we identified several key factors influencing the success of Ỉ pig cloning, specifically in the case of the critically endangered Vietnamese I pig. Our findings demonstrate that the passage number of donor cells exerts a significant effect on the production of cloned pigs. Specifically, donor cells at the 5th and 6th passages proved most suitable for cloned Ỉ pig embryo transfer. Meanwhile, neither the donor cell line nor the sex of the donor cells showed a significant impact on SCNT outcomes. These results provide clear and practical guidance for improving the efficiency of Ỉ pig cloning in Vietnam.

## 1. Introduction

Swine are among the most important livestock species worldwide and are also widely used as animal models in biomedical and biological research. The increasing importance of sustainable livestock production and advances in biomedical research have emphasized the need to conserve livestock genetic diversity and safeguard valuable animal genetic resources [1]. This issue is particularly important for indigenous breeds, which often possess unique adaptive and production-related traits that have been shaped by long-term selection under local environmental conditions. In Vietnam, indigenous pig breeds constitute an important component of the national animal genetic resources and are characterized by several desirable traits, including disease resistance, favorable meat quality, relatively low nutritional requirements, and strong adaptation to local climatic conditions. Nevertheless, a number of indigenous pig breeds have experienced severe population declines or have become extinct, raising concerns about the irreversible loss of unique genetic resources [2].

Among these breeds, the Ỉ pig is considered one of the most threatened indigenous pig breeds in Vietnam. The population of the Ỉ pig was estimated at approximately 675,000 animals in 1991 but declined dramatically to only about 120 animals by 2010. The breed was subsequently classified as endangered by the Food and Agriculture Organization (FAO) in 2007 [3]. The vulnerability of this population has been further exacerbated by disease outbreaks, including the emergence of African swine fever (ASF) in Vietnam in 2019. Given the extremely limited population size and restricted availability of reproductive materials, conventional breeding alone may be insufficient to effectively preserve the genetic diversity of the Ỉ pig. Consequently, complementary reproductive and biotechnological approaches are needed to support the long-term conservation of this valuable genetic resource.

Somatic cell nuclear transfer (SCNT) has emerged as a potential complementary strategy for the conservation and propagation of genetically valuable livestock. Following the birth of Dolly, the first mammal cloned from an adult somatic cell, SCNT has been successfully applied to a range of mammalian species and has attracted considerable interest in livestock production, genetic resource conservation, and biomedical research [4,5]. A major advantage of SCNT for genetic conservation is its ability to generate embryos from somatic cells, thereby providing an opportunity to preserve and propagate genetic material from selected individuals when conventional reproductive materials are limited or unavailable. In pigs, full-term development has been achieved following the transfer of SCNT embryos reconstructed from cryopreserved somatic cells into recipient females [6]. These findings indicate that SCNT may have particular relevance for the conservation of rare or endangered pig breeds, including populations in which the number of reproductively available individuals is severely restricted.

Despite its potential, the practical application of SCNT remains constrained by its relatively low efficiency. In pigs, the proportion of reconstructed embryos that ultimately develop into viable offspring remains low, with reported efficiencies of approximately 1% under certain experimental conditions [7,8]. Improving the developmental competence of SCNT embryos is therefore essential for increasing the feasibility of cloning as a tool for genetic resource conservation. Among the factors affecting SCNT efficiency, donor cells play a central role because their biological characteristics can influence nuclear reprogramming, embryo development, and the subsequent establishment of pregnancy. Previous studies have demonstrated that donor cell characteristics, including passage number, cell line, and donor sex, can significantly affect the developmental competence of SCNT embryos and cloning outcomes in pigs [9]. The characteristics of somatic donor cells, including cell type, cell line, passage number, and donor sex, may influence the developmental competence of porcine SCNT embryos and the efficiency of cloning. Previous studies have reported that different donor cell types and cell lines can affect cloning outcomes, while recent work in the endangered Vietnamese Ỉ pig demonstrated that donor cell passage number had measurable effects on embryo development. However, no significant differences were observed between male- and female-derived donor cells or among different cell lines under the conditions tested [10]. Thus, optimization of donor cell parameters represents an important avenue for improving the efficiency and reproducibility of porcine SCNT.

In Vietnam, several studies have focused on improving the efficiency of SCNT embryo production in the Ỉ pig, including optimization of donor cell preparation and characteristics [10] and improvements in oocyte maturation and embryo culture systems [11]. Van et al. (2024) [10] demonstrated that donor cell passage number, cell line, and donor sex significantly influenced the efficiency of SCNT-derived Ỉ pig embryo production. However, whether these donor cell-related factors exert similar effects on the subsequent production of live cloned Ỉ piglets remains insufficiently understood. This distinction is important because improved embryo production does not necessarily translate into increased efficiency of live offspring production. Moreover, systematic evaluation of donor cell parameters at the level of cloned piglet production is essential for identifying suitable donor cell sources for the conservation of the Ỉ pig.

Therefore, the present study aimed to evaluate the effects of key donor cell characteristics, including passage number, cell line, and donor sex, on the efficiency of cloned Ỉ pig production. The findings may provide further insight into the optimization of donor cell selection for Ỉ pig SCNT and contribute to the development of complementary strategies for the long-term conservation of the Ỉ pig genetic resource.

## 2. Materials and Methods

### 2.1. Animals

All animal experiments in this study were conducted in strict compliance with Vietnamese legislation, and Decision No. 2656/QĐ-BNN-KHCN issued by the Ministry of Agriculture and Rural Development on 15 June 2021 (currently under the Ministry of Agriculture and Environment). All effort was undertaken to minimize animal suffering.

The data analyzed in this study were derived from the production of cloned Ỉ pigs at our laboratory between January 2020 and July 2024. All embryo transfer procedures were performed at the Vietnam Institute of Animal and Veterinary Sciences.

### 2.2. Reagents and Chemicals

All reagents and chemicals used in this study otherwise indicated were purchased from Sigma-Aldrich Inc. (St. Louis, MO, USA). Cell culture dishes were sourced from Corning Invc. (Corning, NY, USA).

### 2.3. Oocyte Collection and In Vitro Maturation (IVM)

Porcine oocyte recovery and in vitro maturation (IVM) were conducted following the procedure established by Van et al. (2021) [11], with the relevant culture conditions described below. Ovaries were collected from 6–8-month-old prepubertal Landrace × Large White (LW) gilts at a local slaughterhouse and transported to the laboratory within 3–5 h. During transportation, the ovaries were maintained at 35–37 °C in saline containing 0.1 mg/mL streptomycin sulfate and 100 units/mL penicillin G potassium (Sigma-Aldrich). For COC recovery, follicles measuring ≥2 mm in diameter were scraped into TALP-HEPES medium supplemented with penicillin G potassium (100 units/mL) and streptomycin sulfate (0.1 mg/mL; Sigma-Aldrich). The recovered cumulus–oocyte complexes (COCs) were subsequently inspected and selected using a stereomicroscope.

IVM was performed in porcine oocyte medium (POM) formulated according to Yoshioka et al. (2008) [12] and supplemented with 10 ng/mL epidermal growth factor (EGF; Sigma-Aldrich), 10 IU/mL equine chorionic gonadotropin (eCG; Serotropin, ASKA Pharmaceutical Co., Ltd., Tokyo, Japan), and 10 IU/mL human chorionic gonadotropin (hCG; Puberogen, Novartis Animal Health, Tokyo, Japan), as previously described by Van et al. (2021) [11]. During the first 22 h of maturation, COCs were cultured in the presence of 1 mM dibutyryl cyclic AMP (dbcAMP; Sigma-Aldrich). Groups of 30–50 COCs were placed in four-well culture dishes (Nunc MultiDishes, Thomas Scientific, Swedesboro, NJ, USA), with each well containing 500 µL of IVM medium covered with mineral oil (Sigma-Aldrich). The cultures were maintained at 39 °C under a gas mixture of 5% CO_2_, 5% O_2_, and 90% N_2_. After the initial culture period, the COCs were transferred to fresh maturation medium lacking dbcAMP and incubated for a further 22–24 h under identical temperature and gas conditions.

### 2.4. Donor Cells Preparation

Donor cells were prepared according to the protocol of Van et al. (2021) [11]. Briefly, marginal ear tissue was obtained by ear-punch biopsy from five 1-month-old Ỉ gilts (IDs: 6004, 9154, 9155, 9156, and 9157) and one 1-month-old Ỉ boar (ID: 6002). The collected tissues were cultured in Dulbecco’s Modified Eagle Medium (DMEM; Sigma-Aldrich) supplemented with 10% fetal bovine serum (FBS) and maintained at 37 °C in a humidified atmosphere containing 5% CO_2_. Upon reaching ≥80% confluency, cells were detached with 0.25% Trypsin-EDTA (Sigma-Aldrich) and expanded through three passages following the method described by Freshney (2000) [13]. Cells at the 3rd passage were subsequently cryopreserved in a medium containing 70% DMEM, 20% FBS, and 10% dimethyl sulfoxide (DMSO; Sigma-Aldrich), and stored in liquid nitrogen [14].

To evaluate the effect of donor cell passage number on embryo transfer efficiency in Ỉ pigs, donor cells at the 4th and 7th passages were cultured until reaching at least 80% confluency and subsequently used for somatic cell nuclear transfer (SCNT). Donor cells at the 4th and 7th passages were examined under a microscope (Axiovert 200M, Carl Zeiss, Oberkochen, Germany). Forty-eight hours before nuclear transfer, donor cells were cultured in medium containing 0.2% fetal bovine serum (FBS) to induce cell cycle synchronization. Approximately 30 min before nuclear transfer, donor cells were harvested using 0.25% (*w*/*v*) trypsin–0.02 mM EDTA (Sigma-Aldrich), followed by washing and resuspension in HEPES-buffered Tissue Culture Medium 199 (TCM-199).

### 2.5. SCNT and Embryo Culture

SCNT embryos were produced using a zona-free procedure with minor modifications to previously reported protocols [15,16,17,18]. After 40–42 h of in vitro maturation, cumulus cells were removed by vortexing the oocytes in TALP-HEPES medium supplemented with 1 mg/mL hyaluronidase (Sigma-Aldrich). Oocytes with an extruded polar body were subsequently treated with 0.5% Pronase (Sigma-Aldrich) in TALP-HEPES for 3–6 min to remove the zona pellucida.

**Figure 1:**
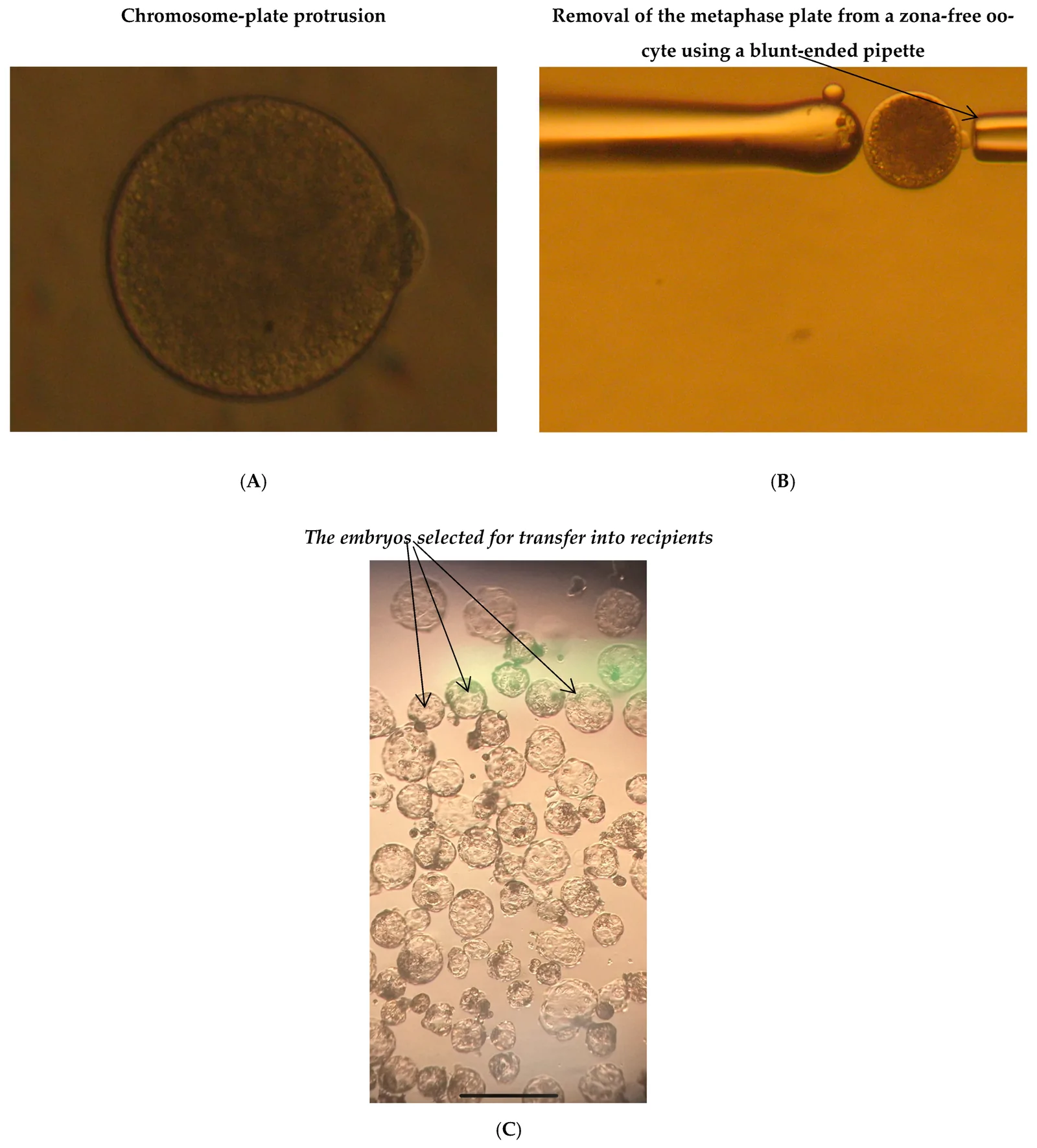
(**A**) Zona pellucida–free oocytes exhibiting chromosome-plate protrusion were obtained following treatment with 4 μM demecolcine in PZM3 medium supplemented with 2.5% FBS (eyepiece × objective is 20×/0.4). (**B**) Removal of the metaphase plate from a zona-free oocyte using a blunt-ended pipette (eyepiece × objective is 10×/0.25). (**C**) Zona pellucida–free cloned Ỉ pig blastocysts were generated using SCNT technique (eyepiece × objective is 5×/0.12).

Following zona removal, the oocytes were rinsed in TALP-HEPES containing fetal bovine serum (FBS) and transferred to PZM3 medium [19] supplemented with 2.5% FBS until enucleation. To facilitate chromosome visualization and removal, zona-free oocytes were exposed to 4 μM demecolcine (Sigma-Aldrich) in PZM3 containing 2.5% FBS for 20–40 min. Oocytes exhibiting a characteristic protrusion (Figure 1A) were subjected to enucleation in TALP-HEPES supplemented with 7.5 μg/mL cytochalasin B (Sigma-Aldrich). A blunt-ended pipette together with a closed holding pipette was used to remove the metaphase chromosomes (Figure 1B).

The resulting zona-free cytoplasts were briefly rinsed in HEPES-buffered TCM-199 containing 300 μg/mL phytohemagglutinin (Sigma-Aldrich) and immediately positioned over an individual donor cell settled at the bottom of a 100-μL TALP-HEPES droplet. This procedure facilitated close contact between the cytoplast and donor cell. The resulting couplets were then washed for 2–3 min in fusion medium consisting of 0.3 mM mannitol, 0.05 mM CaCl_2_·2H_2_O, 0.1 mM MgSO_4_·7H_2_O, and 25 mg/mL polyvinyl alcohol (PVA). The couplets were transferred to a fusion chamber containing 2 mL of pre-warmed fusion medium and subjected to two direct-current (DC) pulses of 70 V for 30 μs using an electro-cell fusion system (LF 101, Nepa Gene Co., Ltd., Chiba, Japan). After confirmation of fusion, the couplets were incubated for 2 h at 38.5 °C in a humidified atmosphere containing 5% CO_2_ and 5% O_2_. Activation was subsequently induced with a single DC pulse of 65 V for 80 μs. The activated embryos were maintained for an additional 3 h in PZM3 supplemented with 7.5 μg/mL cytochalasin B under the same culture conditions.

Embryo culture was performed using a modified Well-of-the-Well (WOW) system as described by Vajta et al. (2000) [20]. Briefly, each 35-mm Petri dish (Corning, NY, USA) contained 20 microwells, each covered with a 100-μL microdrop of PZM3 medium and overlaid with mineral oil. One SCNT embryo was placed in each microwell and cultured at 38.5 °C in a humidified atmosphere containing 5% CO_2_ and 5% O_2_. The day of activation was designated as Day 0. On Day 5, FBS was added to the culture medium at a final concentration of 10% (*v*/*v*). Embryos that developed to the morula or blastocyst stage (Figure 1C) were evaluated microscopically using an Axiovert 200M microscope (Carl Zeiss, Oberkochen, Germany) and collected on Days 5 and 6.

### 2.6. Embryo Transfer

Landrace × Yorkshire gilts weighing 100–120 kg with one or two previous estrous cycles were used as recipients for SCNT embryo transfer. Estrous synchronization was induced by intramuscular injection of 1000 IU equine chorionic gonadotropin (eCG; ZENOAQ, Koriyama, Fukushima, Japan), followed 72 h later by 1500 IU human chorionic gonadotropin (hCG; ZENOAQ, Koriyama, Fukushima, Japan). Compacted morulae and blastocysts (Figure 1B) derived from SCNT were transferred to the uterine horns on Day 5 after the onset of standing estrus. Embryo transfer was conducted surgically through a mid-ventral laparotomy under general anesthesia. Embryos were suspended in NCSU-37 medium supplemented with 5 mg/mL bovine serum albumin (BSA) and 20 mM HEPES, without FBS, as previously described [21]. Recipient pregnancy was evaluated 28 days after embryo transfer using transabdominal ultrasonography with a BestScan^®^S3 Wireless ultrasound system (BMV Technology Co., Ltd., Shenzhen, China).

### 2.7. Experimental Design

In each Experiment, the percentage of piglets born was determined by dividing the number of piglets by the number of transferred embryos and multiplying the result by 100%.

*Experiment 1. The effect of donor cell passage number on the efficiency of cloned Ỉ pig embryo transfer.*

This experiment was designed to assess the effect of donor fibroblast passage number on the developmental and embryo transfer efficiency of cloned Ỉ pig embryos. Fibroblasts obtained from a single Ỉ pig (ID 6004) at the 4th to 7th passages were prepared and maintained as described above in DMEM supplemented with 0.2% (*v*/*v*) FBS. SCNT embryos were reconstructed using these Ỉ pig fibroblasts and cytoplasts derived from LW oocytes matured in a defined POM system, followed by culture under serum-deprived conditions. The reconstructed embryos were cultured in PZM3 supplemented with FBS until Day 5, with the day of SCNT designated as Day 0. Embryos were transferred to surrogate recipients at either the compacted morula stage on Day 5 or the blastocyst stage on Day 6, following the procedure described above.

*Experiment 2. The effect of sex of donor cells on the efficiency of cloned Ỉ pig embryo transfer*

This experiment aimed to evaluate the impact of donor cell sex on the efficiency of cloned Ỉ pig embryo transfer. As previously outlined, LW oocytes obtained from a slaughterhouse were subjected to IVM using POM. Female and male Ỉ pig fibroblast of 2 different individuals (9154 and 6002, respectively) selected from the cell passage associated with the highest pregnancy rate in *Experiment 1* were introduced into enucleated mature oocytes. Embryos derived from either female or male donor cells were transferred into surrogate sows at two developmental stages: the compacted morula stage on Day 5 or the blastocyst stage on Day 6, following established protocols.

*Experiment 3. The effect of cell lines on the efficiency of cloned Ỉ pig embryo transfer*

This experiment aimed to evaluate the influence of cell lines on the efficiency of cloned Ỉ pig embryo transfer. SCNT embryos were generated using three distinct cell lines of either female or male Ỉ fibroblasts, selected based on the highest pregnancy rates observed in *Experiment 2*, as previously described. Embryos derived from these donor cells were transferred into surrogate sows at two embryonic stages: the compacted morula stage on Day 5 and the blastocyst stage on Day 6, in accordance with established protocols.

### 2.8. Statistical Analysis

Categorical data were presented as frequencies (n) and percentages (%). Differences among the experimental groups were assessed using the chi-square test. A two-sided *p* < 0.05 was considered statistically significant.

## 3. Results

### 3.1. The Effect of Donor Cell Passage on the Efficiency of Cloned Ỉ Pig Embryo Transfer

**Figure 2:**
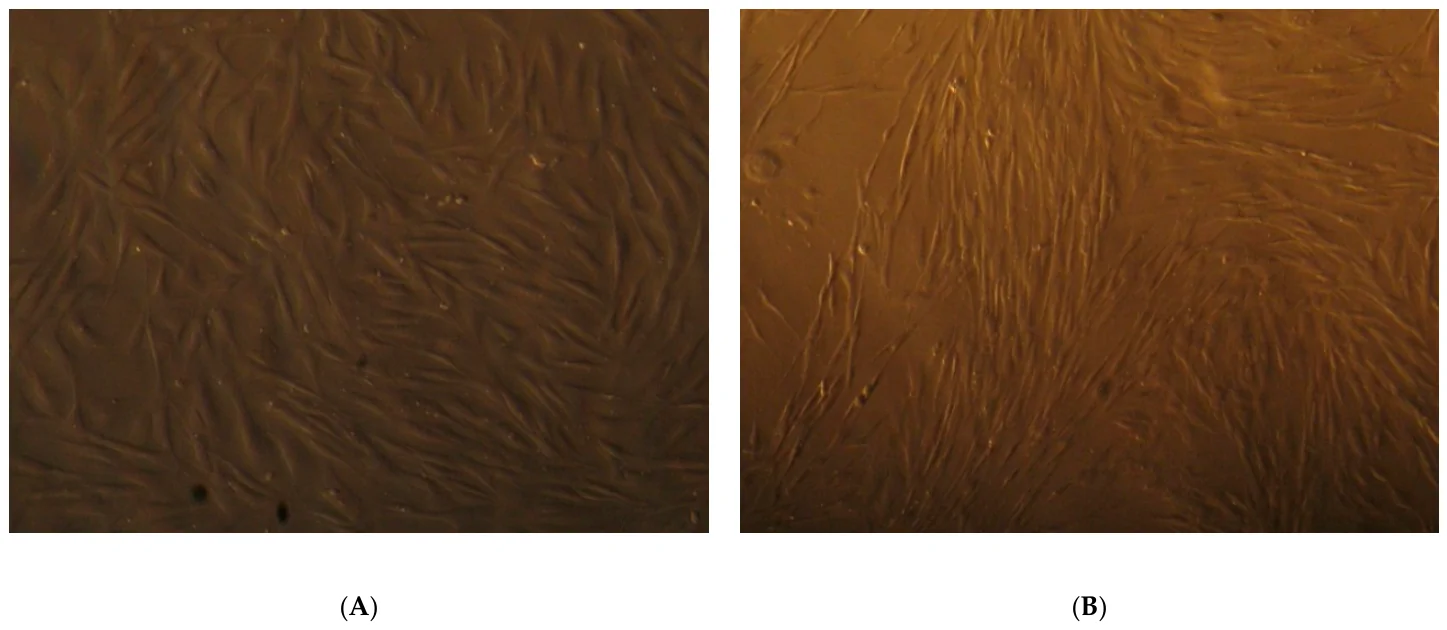
(**A**) Ỉ fibroblasts at the 5th passage were cultured in DMEM supplemented with 10% FBS (eyepiece × objective is 10×/0.25). (**B**) Ỉ fibroblasts at the 7th passage were cultured in DMEM supplemented with 10% FBS (eyepiece × objective is 5×/0.12).

Our findings indicate that Ỉ pig fibroblasts from individual 6004 at the 5th (Figure 2A) and 6th passages exhibited significantly higher rates of pregnancy, farrowing, and piglet production compared to those at the 4th and 7th passages (Figure 2B) (Table 1, *p* < 0.05).

There was no significant difference in the pregnancy, farrowing rates and piglet production between the 5th and 6th passages (Table 1, *p* > 0.05). Ỉ fibroblasts at the 5th and 6th passages are regarded as suitable for the transfer of cloned Ỉ pig embryos.

### 3.2. The Effect of Sex of Donor Cells on the Efficiency of Cloned Ỉ Pig Embryo Transfer

Based on the findings from *Experiment 1*, *Experiment 2* used male and female Ỉ pig fibroblasts derived from two distinct individuals 9154 (female) and 6002 (male) at the 5th passage.

**Figure 3:**
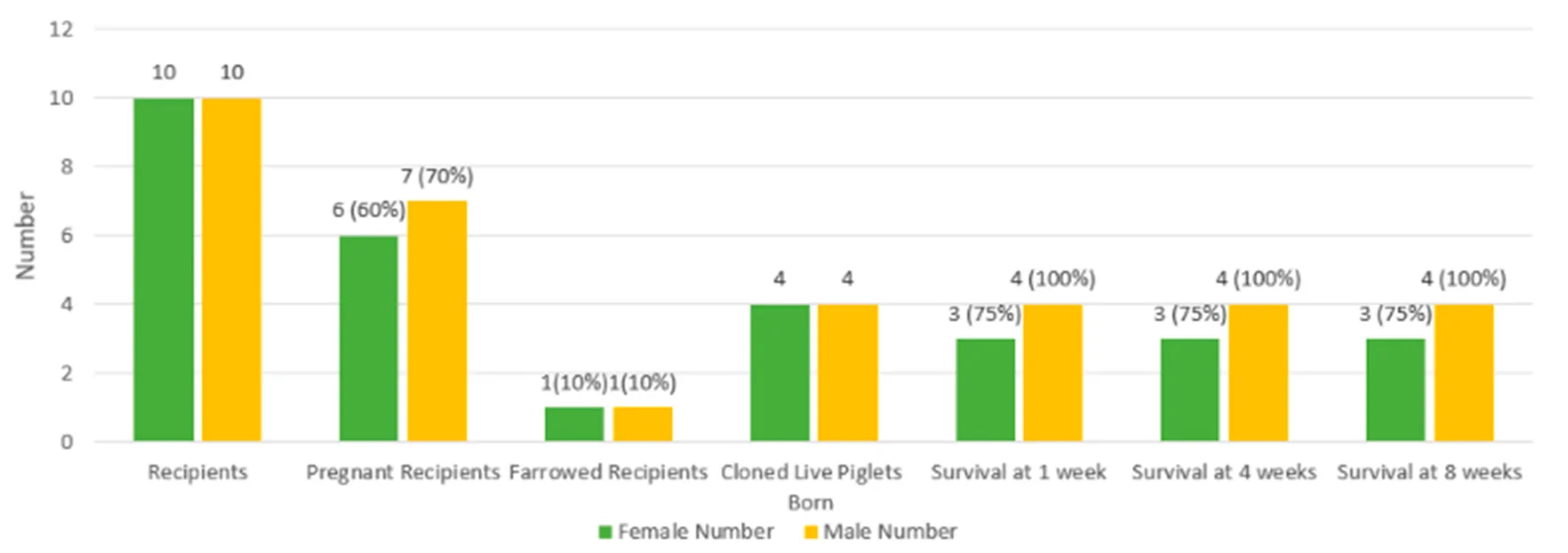
The effect of sex of donor cells on the efficiency if cloned Ỉ pig embryo transfer. The percentage of surviving piglets was calculated as the number of piglets surviving at each time point divided by the total number of piglets born, multiplied by 100.

**Figure 4:**
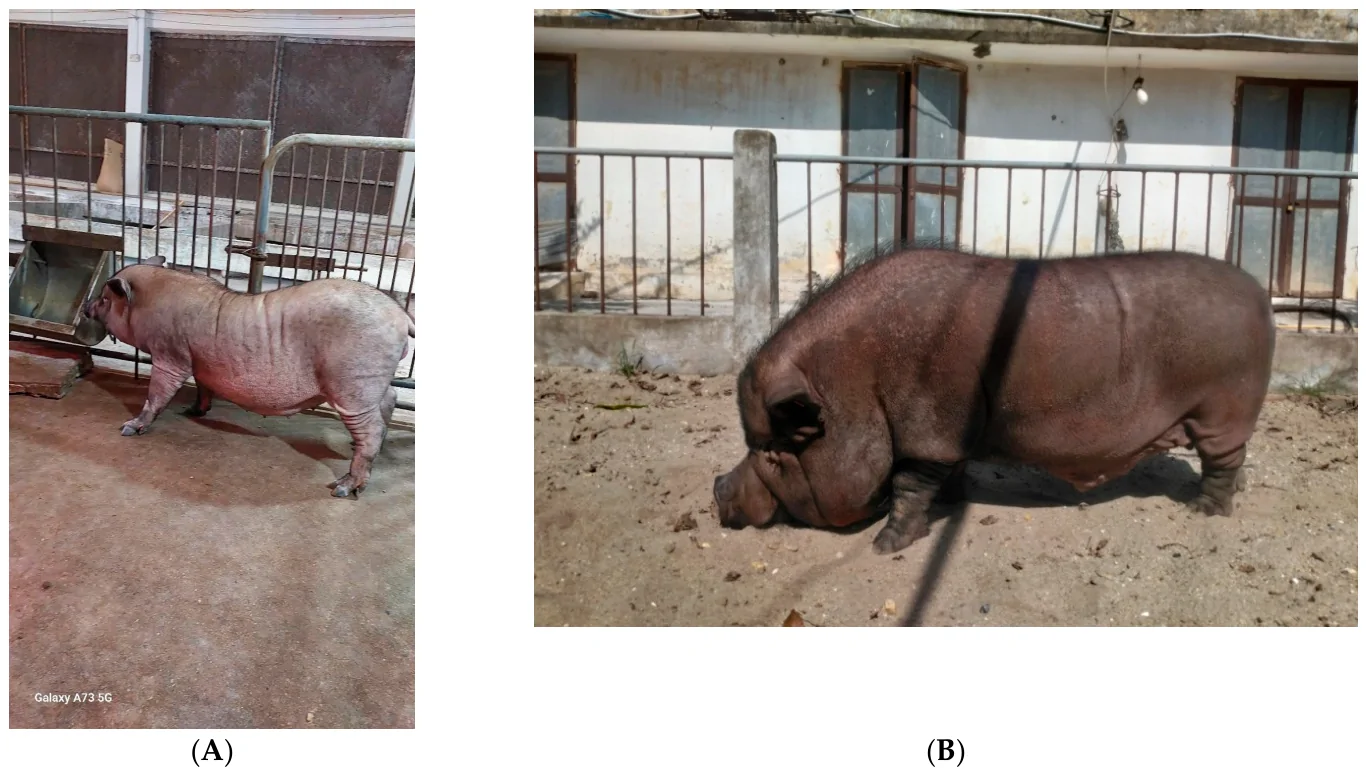
(**A**). Cloned female Ỉ pig was generated using the SCNT technique. (**B**) Cloned male Ỉ pig was generated using the SCNT technique.

The outcomes, summarized in Figure 3, revealed no statistically significant differences in the pregnancy, farrowing rates, and piglet production between the male and female groups (Figure 3, *p* > 0.05). In the both male and female groups, only a single pregnant recipient carried the pregnancy to term. The findings from Figure 3 indicate that the sex of donor cells do not affect the cloned Ỉ pig production efficiency (Figure 4A,B).

### 3.3. The Effect of Cell Lines on the Efficiency of Cloned Ỉ Pig Embryo Transfer

**Figure 5:**
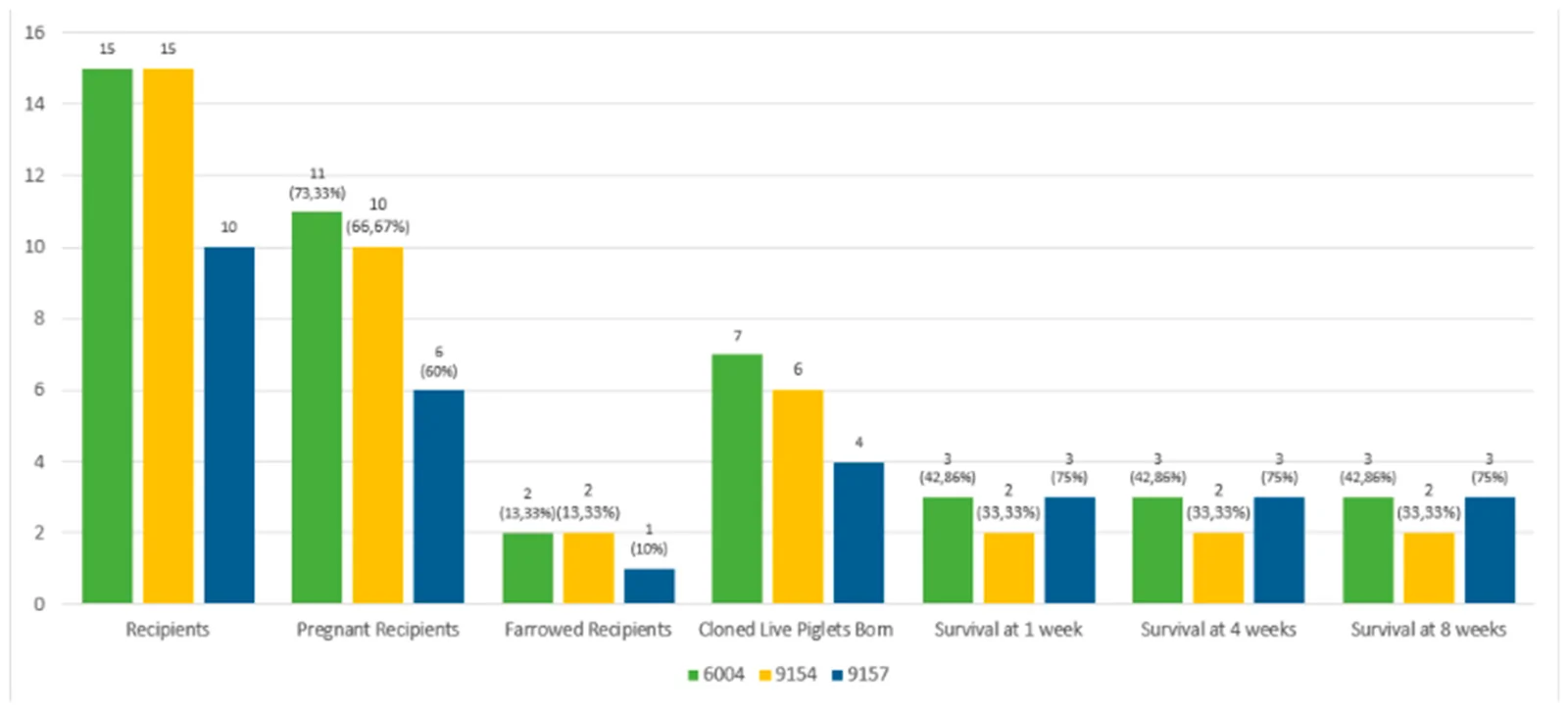
The effect of cell lines on the efficiency of cloned Ỉ pig embryo transfer. The percentage of surviving piglets was calculated as the number of piglets surviving at each time point divided by the total number of piglets born, multiplied by 100.

Building upon the findings of *Experiment 2*, *Experiment 3* employed fibroblast cells derived from three individual female Ỉ pigs (IDs: 6004, 9154, and 9157), all at the 5th passage. The outcomes of this experiment are presented in Figure 5.

Statistical analysis revealed no significant differences in pregnancy rates, farrowing rates, or piglet yield among the three donor cell lines (Figure 5, *p* > 0.05). The survival rates of live cloned Ỉ piglets at one week post-birth from three individual female Ỉ pigs 6004, 9154, and 9157 were 42.86%, 33.33%, and 75%, respectively; however, these differences were not statistically significant (*p* > 0.05). These results suggest that the cell lines does not influence the efficiency of cloned Ỉ pig production.

## 4. Discussion

The efficiency of animal cloning by the SCNT technique is influenced by a multifaceted interaction of factors, among which donor cell characteristics play a important role. This study examined donor cell related factors including passage number, sex, and cell line to assess their influence on the success rate of cloned Ỉ pig embryo transfer, with the aim of improving the outcomes of SCNT-mediated Ỉ pig cloning in Vietnam.

The influence of cell passage number on the efficiency of cloned animal production continues to be a topic of scientific debate. Kubota et al. (2000) [22] reported that long-term culture of donor cells yielded better outcomes than short-term culture. In contrast, Rho et al. (2000) [23] found that donor cells at early passages were more effective than those at later passages. Zhang et al. (2009) [24] demonstrated that donor cells at 3rd to 6th passages yielded significantly higher blastocyst formation rates compared to those at 1st to 2nd and 7th to 10th passages. Van et al. (2024) [10] reported that using donor cells at the 5th or 6th passage significantly increased the blastocyst formation rate in cloned embryos. Additionally, Wells et al. (1999) [25] observed no significant differences in the developmental potential of SCNT embryos based on the number of donor cell passages.

The results of *Experiment 1* demonstrated that the passage number of donor cells had a significant influence on the efficiency of cloned Ỉ pig embryo transfer. Under the conditions of this study, donor cells at passage numbers below 5 or above 6 were identified as optimal for the production of cloned pigs via SCNT technique. Our findings are consistent with those reported by Jin et al. (2019) [26]. Jin et al. (2019) [26] demonstrated that the cloning efficiency of piglets was higher when porcine fibroblasts at the 5th or 6th passage were used, compared to cells at earlier than the 5th or beyond the 6th passage. Importantly, fibroblasts with more than 6th passage failed to produce cloned piglets. Therefore, Jin et al. (2019) [26] limited the use of porcine fibroblasts more than the 6th passage from use as donor cells in SCNT for cloned pig production. Contrary to our findings, Kubota et al. (2000) [22] and Miyoshi et al. (2003) [27] reported that the optimal range of donor cell passages for cloned bovine embryo production lies between the 10th and 15th passages. Notably, Miyoshi et al. (2003) [27] identified the 15th passage as particularly suitable for cattle cloning. Discrepancies among studies may be attributed to variations in donor cell origin and laboratory-specific experimental protocols. In the current study, porcine fibroblasts were utilized, whereas Kubota et al. (2000) [22] and Miyoshi et al. (2003) [27] employed bovine fibroblasts.

The present study demonstrated that the passage number of donor cells was associated with differences in the developmental competence of SCNT embryos. Notably, donor cells at the 5th and 6th passages resulted in relatively favorable developmental outcomes compared with cells at earlier or later passages. Although these findings suggest that donor-cell passage is an important factor influencing SCNT efficiency, the biological mechanisms underlying this passage-dependent effect remain unclear. One possible explanation is that donor cells may undergo progressive changes in their cellular and epigenetic characteristics during in vitro culture, resulting in a passage-dependent variation in their suitability for nuclear reprogramming.

The present study demonstrated that donor-cell passage number was associated with differences in the developmental competence of SCNT embryos. Within the passage range examined, donor cells at the 5th and 6th passages produced relatively favorable developmental outcomes, including the successful production of cloned pigs, whereas cells at the 4th and 7th passages showed lower overall performance. These findings suggest that donor-cell passage may influence the suitability of fibroblasts as nuclear donors. However, the relationship does not appear to be simply linear, as both relatively early and later passages showed less favorable outcomes than the intermediate passages examined in this study. Thus, the 5th and 6th passages may represent a potentially favorable culture window under the specific conditions used for donor-cell preparation and SCNT.

The biological basis of this apparent passage-dependent effect remains unclear. Passage number itself may not directly determine the developmental competence of donor cells but may instead reflect progressive changes in their physiological and molecular state during in vitro culture. Somatic cells undergo repeated proliferation during culture, and this process can be accompanied by alterations in epigenetic regulation, cell-cycle distribution, proliferative capacity, cellular stress responses, and genomic stability. Because successful SCNT requires extensive reprogramming of the transferred somatic nucleus by the recipient oocyte, variation in these cellular properties could affect the ability of donor nuclei to undergo appropriate nuclear reprogramming and subsequently support embryo development.

Epigenetic regulation is particularly relevant in this context. During in vitro culture, donor cells may undergo changes in DNA methylation, histone modifications, chromatin organization, and RNA-mediated regulatory mechanisms, including m6A modification [28]. Such alterations may influence chromatin accessibility and the expression of genes involved in early embryonic development, thereby affecting the ability of the transferred nucleus to respond to the reprogramming environment of the oocyte. It is therefore conceivable that donor cells at the 5th and 6th passages may possess an epigenetic configuration that is relatively compatible with nuclear reprogramming, whereas prolonged culture may progressively introduce epigenetic alterations that reduce developmental competence. However, this interpretation remains speculative because epigenetic characteristics were not directly assessed at each passage in the present study.

Cellular aging provides another possible explanation for the lower performance observed at later passages. Extended in vitro culture and repeated cell divisions can increase cellular stress and may be associated with reduced proliferative capacity, senescence, oxidative stress, and accumulation of genetic or epigenetic alterations [29]. These changes may modify chromatin organization and cellular function and, consequently, affect the reprogramming competence of donor nuclei. In contrast, cells at very early passages may differ in their adaptation to the in vitro culture environment and in their physiological state. Thus, the relatively favorable outcomes at the 5th and 6th passages may reflect an intermediate stage at which donor cells retain sufficient proliferative capacity and cellular integrity while avoiding some of the detrimental changes associated with prolonged culture. This interpretation should nevertheless be considered a possible explanation rather than a demonstrated mechanism.

The cell-cycle state of donor cells may also contribute to passage-dependent differences in SCNT efficiency. The developmental competence of reconstructed embryos depends, in part, on appropriate coordination between the donor nucleus and the recipient oocyte. Changes in proliferation kinetics during culture may alter the distribution of donor cells across different cell-cycle stages, potentially affecting the physiological state of the nucleus at the time of nuclear transfer. Although donor cells in the present study were subjected to the same general experimental procedures, cell-cycle distribution and synchronization efficiency were not directly evaluated across passages. Consequently, it cannot be determined whether the more favorable outcomes observed at the 5th and 6th passages were associated with a particular cell-cycle profile.

The possibility of passage-related changes in genomic stability should also be considered. Repeated cell division and prolonged culture may increase cellular stress and the accumulation of genetic or chromosomal abnormalities, whereas cells at earlier passages may have different characteristics resulting from their initial adaptation to culture conditions. In addition, prolonged culture may affect the regulation of imprinted genes, which are particularly important for normal embryonic and fetal development [30]. These processes could collectively contribute to a decline in donor-cell suitability at later passages. Rather than acting independently, epigenetic alterations, cellular aging, cell-cycle changes, and genomic instability may interact and collectively influence the capacity of donor nuclei to undergo successful reprogramming.

The effect of donor-cell passage number on SCNT efficiency has been inconsistent among previous studies. Some studies have reported a decline in developmental competence with increasing passage number [26], whereas others have found no significant association between passage number and cloning efficiency [27]. Such discrepancies may arise from differences in donor breed or genotype, tissue origin and characteristics of fibroblasts, culture conditions, passage ranges, and procedures used for oocyte maturation, nuclear transfer, embryo activation, and recipient management. These differences indicate that the effect of passage number may be context-dependent rather than a universal characteristic of donor cells. Accordingly, the favorable performance observed at passages 5 and 6 in the present study should be interpreted specifically within the experimental conditions and donor-cell system used here.

Importantly, the present study was designed primarily to evaluate the practical relationship between donor-cell passage and the efficiency of cloned pig production rather than to identify the molecular mechanisms responsible for passage-dependent differences. Within the passage range examined, fibroblasts retained sufficient developmental competence to support embryo development and the production of cloned pigs, while the successful production of cloned pigs was observed only when donor cells from the fifth and sixth passages were used. Although this finding suggests that these passages may provide a useful operational window for donor-cell preparation, the limited sample size and lack of direct characterization of donor-cell properties warrant cautious interpretation. In particular, the present data cannot establish whether the apparent advantage of the 5th and 6th passages results from epigenetic status, cellular senescence, cell-cycle characteristics, genomic stability, or a combination of these factors.

Further studies should therefore examine donor cells longitudinally across passages using complementary cellular and molecular approaches, including cell-cycle analysis, assessment of senescence and proliferation, evaluation of DNA damage and genomic stability, and characterization of DNA methylation and other epigenetic modifications. Linking these donor-cell characteristics with subsequent SCNT developmental outcomes would help determine whether an identifiable cellular or molecular state underlies the favorable performance of intermediate-passage cells. Such investigations would provide a mechanistic basis for defining an optimal donor-cell passage range and may further improve the reproducibility and efficiency of SCNT-based cloned pig production.

Subsequently, we evaluated the cloning efficiency of Ỉ piglets using Ỉ fibroblasts derived from both female and male donors. Based on the findings from *Experiment 1*, fibroblasts derived from two distinct individuals, 9154 (female) and 6002 (male), at the 5th passage were utilized as donor cells. Although the female group showed lower pregnancy rates and proportions of cloned live piglets born compared with the male group, these differences were not statistically significant (60% vs. 70% and 0.3% vs. 0.4%, respectively; *p* > 0.05; Figure 3). The pregnancy rates were 60% (6/10) and 70% (7/10) for recipients receiving embryos reconstructed from female and male donor cells, respectively, whereas the farrowing rate was 10% (1/10) in both groups. Each farrowed recipient produced four live cloned piglets, and all piglets survived to 8 weeks of age. Thus, no apparent difference was observed in reproductive outcomes or early survival of cloned piglets according to donor-cell sex. However, because only one recipient in each group farrowed, these observations are descriptive and have limited statistical power. Therefore, further studies with larger sample sizes are required to validate and further clarify the potential effect of donor-cell sex on cloning efficiency.

In the present study, although the small number of farrowed recipients limited the statistical power, the results indicated that SCNT efficiency was comparable among the donor cell lines, including both male and female donor cell lines. Statistical analysis showed that the donor cell line did not significantly affect the efficiency of cloned Ỉ pig production. These findings suggest that, under the conditions of the present study, both male and female donor cells can be used as nuclear donors for SCNT in Ỉ pigs with comparable cloning efficiency. This finding has important implications for the conservation of rare livestock breeds, particularly Ỉ pigs, as it suggests that cloning may enable the reproduction of both male and female offspring with comparable success.

The present findings diverge from those reported by Yoo et al. (2017) [31], yet they align with the observations of Liu et al. (2016) [32]. According to Yoo et al. (2017) [31] no significant differences in pregnancy rates between cloned piglets derived from male versus female donor cells. However, Yoo et al. (2017) [31] reported that, despite the absence of significant differences in pregnancy and delivery outcomes, piglet production was greater when female fibroblast cells were utilized for nuclear reconstruction compared to male fibroblast cells. In contrast, Liu et al. (2016) [32] demonstrated that embryos reconstructed with fibroblast cells derived from male Boer goats yielded significantly higher kid production rates and a greater proportion of live offspring compared to those reconstructed with female-derived fibroblast cells. The developmental potential of SCNT embryos derived from male and female somatic cells remains insufficiently characterized [33]. The efficiency of producing cloned piglets from male and female fibroblasts range from 0.64% to 0.9% [33]. Despite numerous studies on pig cloning, however, the developmental potential of SCNT embryos derived from male and female somatic cells remains insufficiently characterized, and no clear explanation has yet been established for the sex-related differences in mortality among cloned piglets [31,32]. Future research is therefore essential to improve the likelihood of successful development to term and to enhance the postnatal survival of piglets produced through somatic cell nuclear transfer (SCNT) [31].

Based on the results of *Experiments 1* and *2*, *Experiment 3* employed fibroblasts derived from three female Ỉ pigs (6004, 9154, and 9157) at the 5th passage for cloned Ỉ pig production. The study further evaluated the influence of individual donor cell lines on the efficiency of cloned Ỉ pig embryo transfer. No significant differences were observed in pregnancy rates, farrowing rates, or piglet yield among the three cell lines (Figure 5).

The generation of cloned piglets is a highly complex process influenced by numerous factors at both the cellular and embryonic levels, which may ultimately affect the outcome of embryo transfer. These factors include the intrinsic characteristics of donor cells, cell-cycle status, proliferative capacity, epigenetic status, genetic background, and the ability of donor nuclei to undergo complete reprogramming following nuclear transfer. Nevertheless, empirical evidence regarding the influence of individual donor cell lines on embryo transfer outcomes remains limited, and previous studies have reported inconsistent results.

The present findings differ from those reported by Kühholzer et al. (2001) [34]. In their study involving nine donor cell lines derived from nine individual pigs, only four cell lines successfully established pregnancies, and only one pregnancy progressed to day 90 of gestation. The marked variation among cell lines observed in that study suggests that donor-cell origin or intrinsic cellular characteristics may influence the developmental competence of reconstructed embryos. Such differences may be related to variations in genetic background, cellular proliferation, cell-cycle distribution, epigenetic status, or the ability of individual donor nuclei to respond to the reprogramming environment following SCNT.

In contrast, the three cell lines used in the present study were derived from three female Ỉ pigs and were all used at the same passage (the 5th passage). This relatively standardized experimental design may have reduced variation associated with donor-cell sex, passage number, and culture history, thereby allowing a more direct evaluation of differences among individual cell lines. In addition, all donor cell lines were subjected to the same cell culture, SCNT, embryo culture, and embryo transfer procedures. These standardized conditions may have contributed to the comparable pregnancy and farrowing outcomes observed among the three cell lines. Thus, the absence of significant differences in the present study does not necessarily indicate that donor-cell lineage has no effect on cloning efficiency, but rather suggests that under the standardized conditions used here, the three selected cell lines possessed comparable developmental competence.

Our previous study [10] evaluated several donor cell-related factors affecting the efficiency of cloned pig embryo production, including fibroblast number, donor-cell sex, and cell lineage before SCNT. The findings reported by Van et al. (2024) [10] were consistent with those of the present study, further supporting the possibility that appropriate selection and standardization of donor fibroblasts can minimize variation in SCNT outcomes. The discrepancy between the present findings and those of Kühholzer et al. (2001) [34] may therefore be partly attributable to differences in donor-cell characteristics, genetic background, passage number, culture conditions, SCNT procedures, and recipient management between the studies.

Another possible explanation is that the effect of donor cell line may become more apparent when cell lines with greater differences in cellular or molecular characteristics are compared. In the present study, the three cell lines were selected from healthy female Ỉ pigs and were evaluated at the same passage, which may have limited the biological variation among the donor cells. Therefore, the present results should be interpreted within the specific experimental conditions of this study and should not be generalized to all donor cell lines.

Overall, these findings suggest that, when donor fibroblasts are obtained from healthy animals and maintained under standardized culture conditions, individual cell lines may exhibit comparable competence for supporting cloned embryo development and pregnancy. Nevertheless, because only three cell lines were evaluated and the number of recipients was limited, larger studies involving a greater number of genetically diverse donor animals and cell lines are required to determine whether specific donor-cell characteristics can reliably predict SCNT efficiency and embryo transfer outcomes in Ỉ pigs.

## 5. Conclusions

In this study, we investigated some factors associated with donor cells that influence the efficiency of somatic cell nuclear transfer (SCNT) in Ỉ pigs, including donor cell passage number, sex and cell lines. The present study indicates that donor cell passage number represents a critical factor in improving the efficiency of SCNT in Ỉ pigs. The use of donor cells at 5th or 6th passages may enhance the efficiency of cloning outcomes, whereas donor cell sex or cell lines not affect the efficiency of SCNT in Ỉ pigs. Nevertheless, further investigations are required to clarify other factors that may influence cloning efficiency in Ỉ pigs.

## Figures and Tables

**Table 1:** The effect of donor cell passage number on the efficiency of cloned Ỉ pig embryo transfer.

Cell Passage Number	No. of Transferred Embryos	No. of Recipients	No. of Pregnant Recipients (%)	No. of Farrowed Recipients (%)	No. of Cloned Live Piglets Born (%)			
					Total	1 Week	4 Weeks	8 Weeks
4	1500	15	0 (0) ^a^	0 (0)	0	0 (0)	0 (0)	0 (0)
5	1500	15	11 (73.33) ^b^	2 (13.33)	7	3 (42.86)	3 (42.86)	3 (42.86)
6	1500	15	10 (66.67) ^b^	2 (13.33)	6	3 (50)	3 (50)	3 (50)
7	1500	15	2 (13.33) ^a^	0 (0)	0	0 (0)	0 (0)	0 (0)

Different superscripts (a,b) denote a significant difference in the same column (*p* < 0.05). The percentage of surviving piglets was calculated as the number of piglets surviving at each time point divided by the total number of piglets born, multiplied by 100.

## Data Availability

All data generated or analyzed during this study are included in this published article. Additional datasets are available from the corresponding authors on reasonable request.

## Abbreviations

The following abbreviations are used in this manuscript:

SCNT	Somatic cell nuclear transfer
IVM	In vitro maturation
DMEM	Dulbecco’s Modified Eagle Medium
FBS	Fetal bovine serum
COCs	Cumulus–oocyte complexes
LW	Large White

## References

[1] Rátky J., Egerszegi I., Toth P., Keonuchan S., Nagai T., Kikuchi K., Manabe N., Brüssow K.P. (2013). Saving Genetic Resources of Native Pigs in Occidental and Oriental Countries—Practical Examples of the Characterization and Utilization of Native Pigs in Hungary and Laos. J. Reprod. Dev..

[2] Ba N.V., Arakawa A., Ishihara S., Nam L.Q., Thuy T.T.T., Dinh N.C., Ninh P.H., Cuc N.T.K., Kikuchi K., Lan P.D. (2020). Evaluation of Genetic Richness Among Vietnamese Native Pig Breeds Using Microsatellite Markers. Anim. Sci. J..

[3] Rischkowsky B., Pilling D. (2007). The State of the World’s Animal Genetic Resources for Food and Agriculture.

[4] Niemann H., Lucas-Hahn A. (2012). Somatic cell nuclear transfer cloning: Practical applications and current legislation. Reprod. Domest. Anim..

[5] Park M.R., Yoo J.G., Hur C.G., Sim B.W., Kim M., Seo J., Kim B.W., Cho B.W., Shin T.S., Cho S.K. (2020). Effects of variation in the number and developmental stage of donor embryos and ovulation status of the surrogate mother on the efficiency of pig somatic cell cloning. J. Anim. Reprod. Biotechnol..

[6] Men H., Walters E.M., Nagashima H., Prather R.S. (2012). Emerging Applications of Sperm, Embryo and Somatic Cell Cryopreservation in Maintenance, Relocation and Rederivation of Swine Genetics. Theriogenology.

[7] Miranda M.D.S., Bressan F.F., Zecchin K.G., Vercesi A.E., Mesquita L.G., Merighe G.K.F., King W.A., Ohashi O.M., Pimentel J.R.V., Perecin F. (2009). Serum-starved apoptotic fibroblasts reduce blastocyst production but enable development to term after SCNT in cattle. Cloning Stem Cells.

[8] Liu Y., Li J., Løvendahl P., Schmidt M., Larsen K., Callesen H. (2015). In vitro manipulation techniques of porcine embryos: A meta-analysis related to transfer, pregnancies and piglets. Reprod. Fertil. Dev..

[9] Park K.W., Lai L., Cheong H.T., Cabot R., Sun Q.Y., Wu G., Rucker E.B., Durschi D., Bonk A., Samuel M. (2002). Mosaic gene expression in nuclear transfer derived embryos and the production of cloned transgenic pigs from ear-derived fibroblasts. Biol. Reprod..

[10] Van K.N., Huong V.T.T., Huong L.T.N., Huu X.Q., Au T.H., Hieu T.P., Dat V.L., Huong T.N., Nhung T.T.N., Anh L.T.N. (2024). Optimization of donor cell production for somatic cell nuclear transfer in the critically endangered Vietnamese Ỉ pig. Theriogenology.

[11] Van K.N., Tamas S., Salamone D., Huong V.T.T., Huong L.T.N., Huu X.Q., Au T.H., Hieu T.P., Yen K.T.P., Lan D.P. (2021). Optimization of donor cell cycle synchrony, maturation media and embryo culture system for somatic cell nuclear transfer in the critically endangered Vietnamese Ỉ pig. Theriogenology.

[12] Yoshioka K., Suzuki C., Onishi A. (2008). Defined system for in vitro production of porcine embryos using a single basic medium. J. Reprod. Dev..

[13] Freshney R.I. (2000). Culture of animal cells. A Manual of Basic Techniques.

[14] Guan W.J., Ma Y.H., Zhou X.Y., Liu G.L., Liu X.D. (2005). The establishment of fibroblast cell line and its biological characteristic research in Taihang black goat. J. Agric. Sci. Technol..

[15] Booth P.J., Tan S.J., Reipurth R., Holm P., Callesen H. (2001). Simplification of bovine somatic cell nuclear transfer by application of a zona-free manipulation technique. Cloning Stem Cells.

[16] Oback B., Wiersema A.T., Gaynor P., Laible G., Tucker F.C., Oliver J.E., Miller A.L., Trskie H.E., Wilson K.L., Forsyth J.T. (2003). Cloned cattle derived from a novel zona-free embryo reconstruction system. Cloning Stem Cells.

[17] Vajta G., Lewis I.M., Trounson A.O., Purup S., Maddox-Hyttel P., Schmidt M., Pedersen H.G., Greve T., Callesen H. (2003). Handmade somatic cell cloning in cattle: Analysis of factors contributing to high efficiency in vitro. Biol. Reprod..

[18] Lagutina I., Lazzari G., Duchi R., Colleoni S., Ponderato N., Turini P., Crotti G., Galli C. (2005). Somatic cell nuclear transfer in horse: Effect of oocytes morphology, embryo reconstruction method and donor cell type. Reproduction.

[19] Yoshioka K., Suzuki C., Tanaka A., Anas I.M.K., Iwamura S. (2002). Birth of piglets derived from porcine zygotes cultured in a chemically defined medium. Biol. Reprod..

[20] Vajta G., Peura T.T., Holm P., Pasldi A., Trounson A.O., Callesen H. (2000). New method for culture of zona-included or zona-free embryos: The Well of the Well (WOW) system. Mol. Reprod. Dev..

[21] Kikuchi K., Onishi A., Kashiwazaki N., Iwamoto M., Noguchi J., Kaneko H., Akita T., Nagai T. (2002). Successful piglet production after transfer of blastocysts produced by a modified in vitro system. Biol. Reprod..

[22] Kubota C., Yamakuchi H., Todoroki J., Mizoshita K., Tabara N., Barber M., Yang X. (2000). Six cloned calves producted from adult fibroblast cells after long-term culture. Proc. Natl. Acad. Sci. USA.

[23] Rho S., Shim H., Hwang W.S., Yoon J.T. (2000). In vitro development of green fluorescent protein (GFP) transgenic bovine embryos after nuclear transfer using different cell cycles and passages of fetal fibroblasts. Reprod. Fertil. Dev..

[24] Zhang Y.H., Song E.S., Kim E.S., Cong P., Lee S.H., Lee J.W., Yi Y.J., Park C.S. (2009). Effetcs of donor cell passage, size and type on development of porcine embryos derived from somatic cell nuclear transfer. Asian-Australas. J. Anim. Sci..

[25] Wells D.N., Misica P.M., Tervit H.R. (1999). Production of cloned calves following nuclear transfer with cultured adult mural granulosa cells. Biol. Reprod..

[26] Jin Y., Zhang M., Ju X., Liang S., Xiong Q., Zhao L., Nie X., Hou D., Liu Q., Wang J. (2019). Factors influencing the somatic cell nuclear transfer efficiency in pigs. Front. Agric. Sci. Eng..

[27] Miyoshi K., Rzucidlo S.J., Pratt S.L., Stice S.L. (2003). Improvements in cloning efficiencies may be possible by increasing uniformity in recipient oocytes and donor cells. Biol. Reprod..

[28] Zhang M., Zhai Y., An X., Li Q., Zhang D., Zhou Y., Zhang S., Dai X., Li Z. (2024). DNA methylation regulates RNA m^6^A modification through transcription factor SP1 during the development of porcine somatic cell nuclear transfer embryos. Cell Prolif..

[29] Yun J.H., Kang H.G., Choi E.Y., Jeon S.B., Kim M.J., Jeong P.S., Song S., Kim S.U., Min K.S., Sim B.W. (2025). Lycopene enhances epigenetic reprogramming and zygotic genome activation in the porcine somatic cell nuclear transfer embryos. Sci. Rep..

[30] Young L.E., Sinclair K.D., Wilmut I. (1998). Large offspring syndrome in cattle and sheep. Rev. Reprod..

[31] Yoo J.G., Kim B.W., Park M.R., Kwon D.N., Choi Y.J., Shin T.S., Cho B.W., Seo J., Kim J.H., Cho S.K. (2017). Influences of somatic donor cell sex on in vitro and in vivo embryo development following somatic cell nuclear transfer in pig. Asian-Australas. J. Anim. Sci..

[32] Liu H., Peng H., Hu C., Li X.Y., Zhang J.L., Zheng Z., Zhang W.C. (2016). Effects of donor cells sex on nuclear transfer efficiency and telomere lengths of cloned goats. Reprod. Domest. Anim..

[33] Kurome M., Ishikawa T., Tomii R., Ueno S., Shimada A., Yazama H., Nagashima H. (2008). Production of transgenic and non-transgenic clones in Miniature pigs by somatic cell nuclear transfer. J. Reprod. Dev..

[34] Kühholzer B., Hawley R.J., Lai L., Kolber-Simonds D., Prather R.S. (2001). Clonal lines in different development when used for nuclear transfer in pigs. Biol. Reprod..

